# Supplementary figures and images for: Two stages of bandwidth scaling drives efficient neural coding of natural sounds
Source: PLoS Comput Biol. 2023 Feb 14;19(2):e1010862. doi: 10.1371/journal.pcbi.1010862 (PMC9970106; doi:10.1371/journal.pcbi.1010862)

Fourier Spectra  $\Delta f=30$

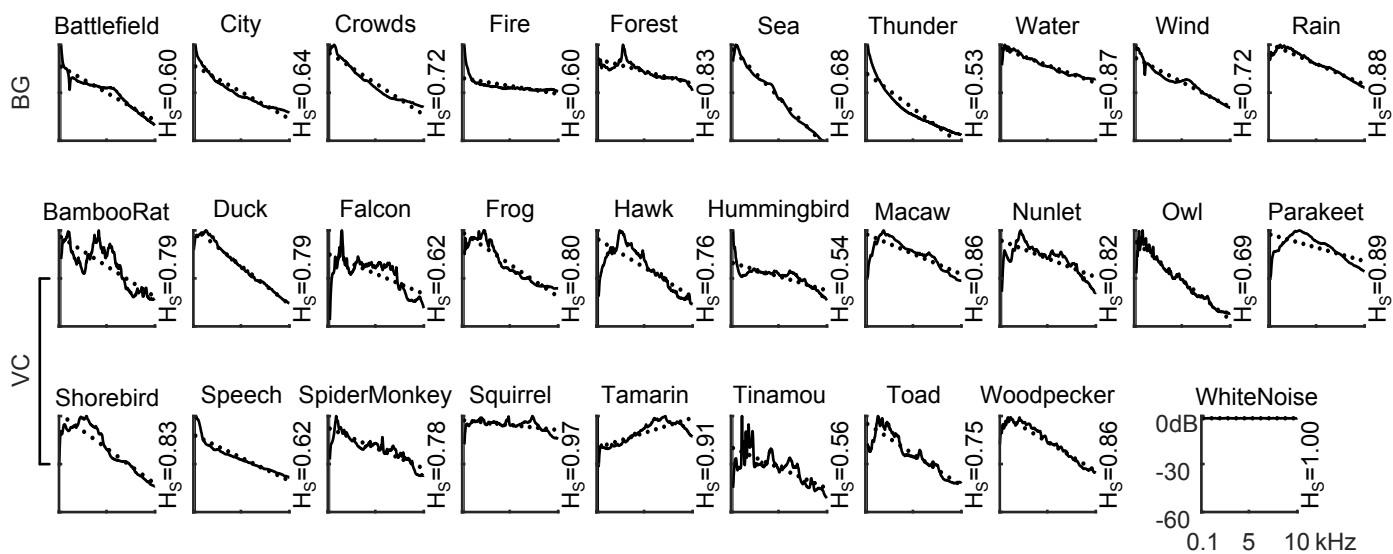

Fourier Spectra  $\Delta f=120$

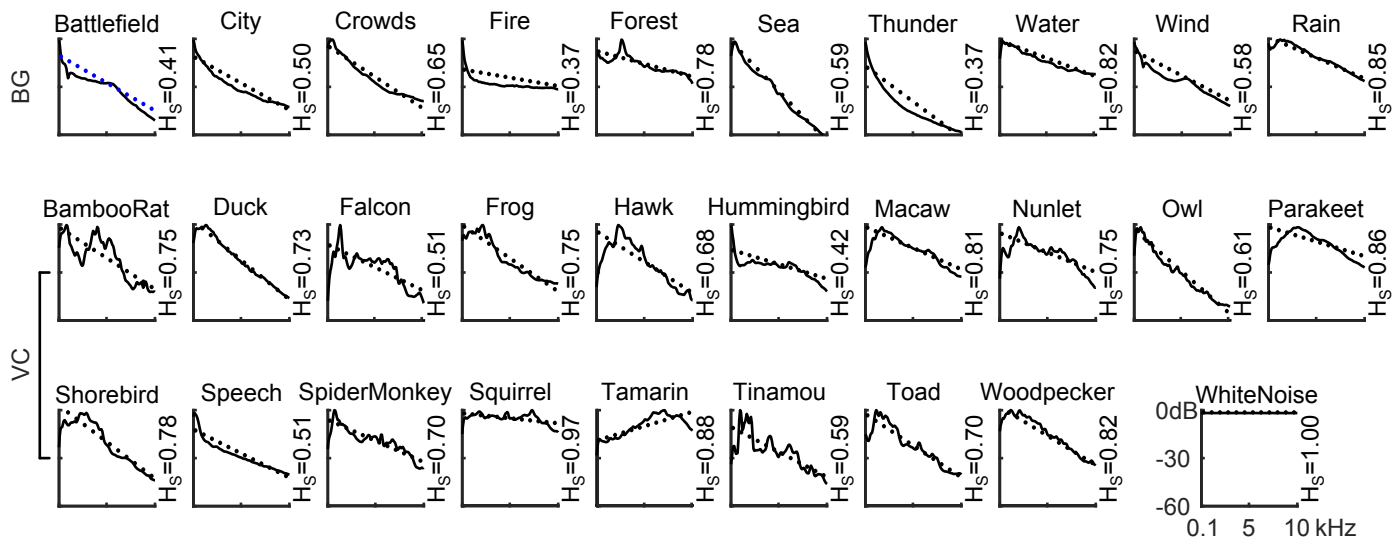

Fourier Spectra  $\Delta f=480$

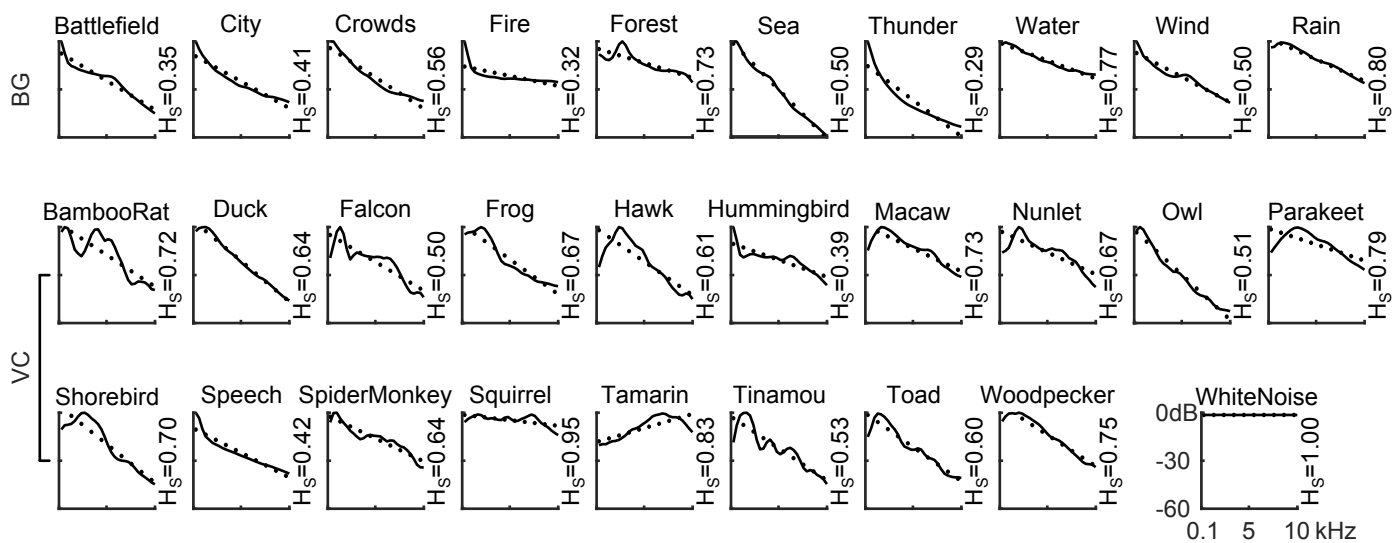

Supplement: S1 Fig — Power spectra for all sound categories are analyzed using the Fourier-based model with resolutions: 30, 120 and 480Hz. (PDF) [file pcbi.1010862.s001.pdf]

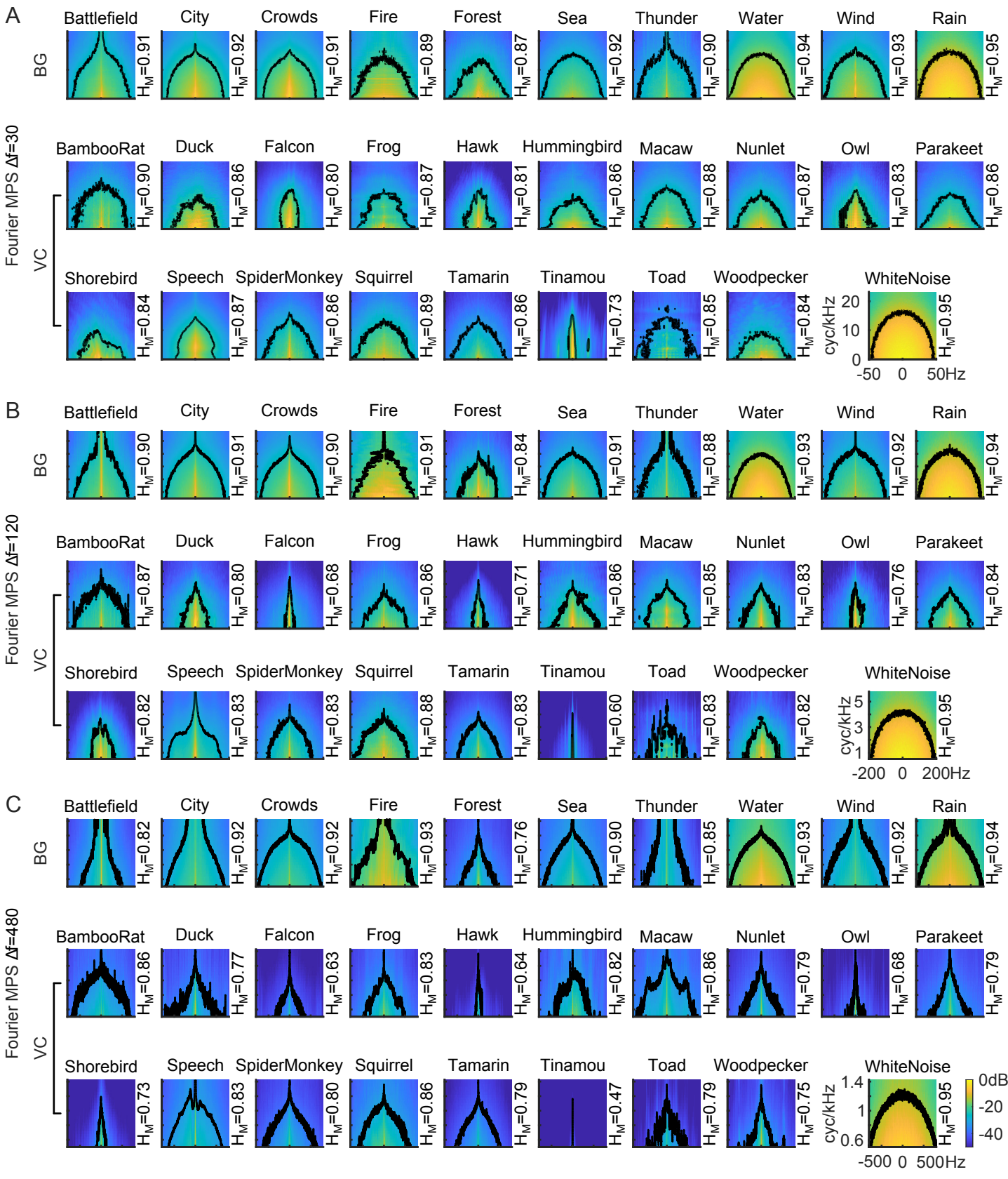

Supplement: S2 Fig — Modulation power spectra for all sound categories are analyzed with the Fourier model with resolutions: 30, 120 and 480Hz. (PDF) [file pcbi.1010862.s002.pdf]

A

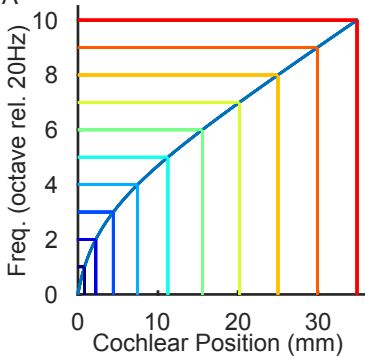

B

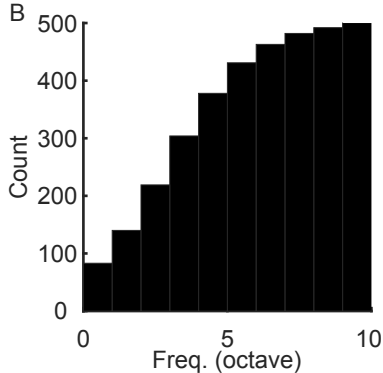

C

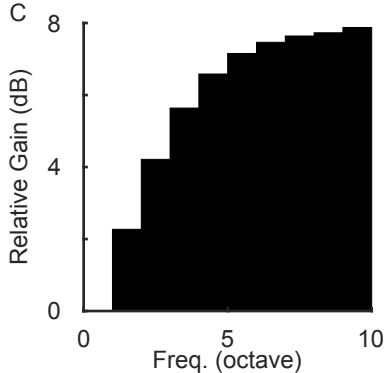

Supplement: S4 Fig — (A) Frequency-position function for the human cochlea proposed by Greenwood [31,43] is broken up into 1 octave segments spanning low (20 Hz, blue) to high (20 kHz, red) frequencies. The lowest octave range (20–40 Hz) spans ~0.8 mm of the cochlear spiral while the highest octave spans ~5 mm. (B) Predicted hair cell count for different frequency ranges (1 octave segments) obtained by assuming 100 hair cells / mm [44]. Hair cell counts increase with increasing frequency resulting in ~5 times as many hair cells per octave for high frequencies. (C) Predicted cochlear output gain of our model for different 1 octave segments arising from hair cell density. The increased hair cell density per octave at high frequencies produces an ~8 dB increase in our model output power relative to the lowest frequencies. (PDF) [file pcbi.1010862.s004.pdf]

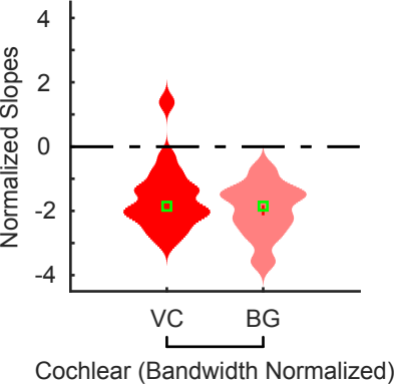

Supplement: S6 Fig — The normalized slope distributions (shown as Violin plots) for vocalization and background sounds exhibit similar trends as for the Fourier power spectrum (compare with Fig 5A). (PDF) [file pcbi.1010862.s006.pdf]
