# Supplementary material for: Two stages of bandwidth scaling drives efficient neural coding of natural sounds
Source: PLoS Comput Biol. 2023 Feb 14;19(2):e1010862. doi: 10.1371/journal.pcbi.1010862 (PMC9970106; doi:10.1371/journal.pcbi.1010862)
Supplement: S3 Fig — Each sound category is plotted as in Fig 7, except that each is normalized to an individual power range and colorscale for visual clarity. (PDF) [file pcbi.1010862.s003.pdf]

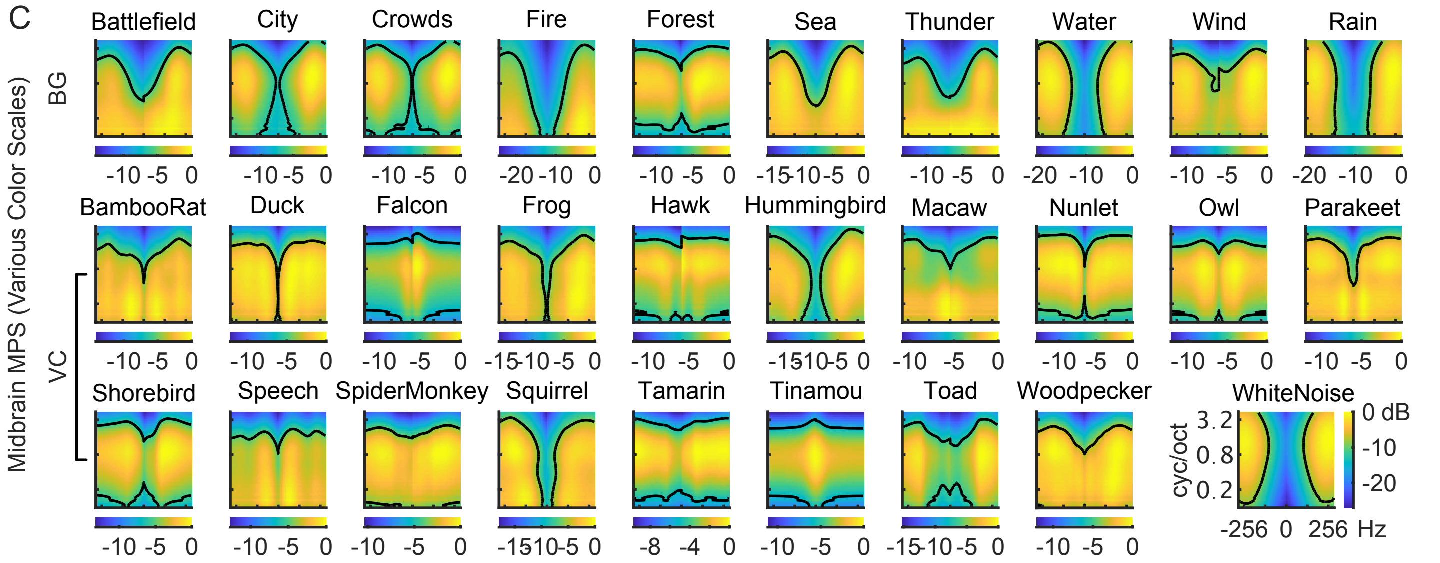
C
Battlefield
City
Crowds
Fire
Forest
Sea
Thunder
Water
Wind
Rain
BG
BambooRat
Duck
Falcon
Frog
Hawk
Hummingbird
Macaw
Nunlet
Owl
Parakeet
Shorebird
Speech
SpiderMonkey
Squirrel
Tamarin
Tinamou
Toad
Woodpecker
WhiteNoise
VC
Midbrain MPS (Various Color Scales)
cyc/oct
3.2
0.8
0.2
-256 0 256 Hz
0 dB
-10
-20
