# Supplementary material for: Two stages of bandwidth scaling drives efficient neural coding of natural sounds
Source: PLoS Comput Biol. 2023 Feb 14;19(2):e1010862. doi: 10.1371/journal.pcbi.1010862 (PMC9970106; doi:10.1371/journal.pcbi.1010862)
Supplement: S5 Fig — The cochlear spectrum of natural sounds (outputs of the cochlear model) shown in Fig 4 (panels B) were normalized by the cochlear filter bandwidths. This provides the cochlear output power per Hz. The results for each natural sound closely resemble the Fourier spectrum of Fig 4A suggesting that flatting of the cochlear spectrum observed in Fig 4A arises because of the cochlear bandwidth scaling. Dotted lines correspond to the linear regression fits for each natural sound category. (PDF) [file pcbi.1010862.s005.pdf]

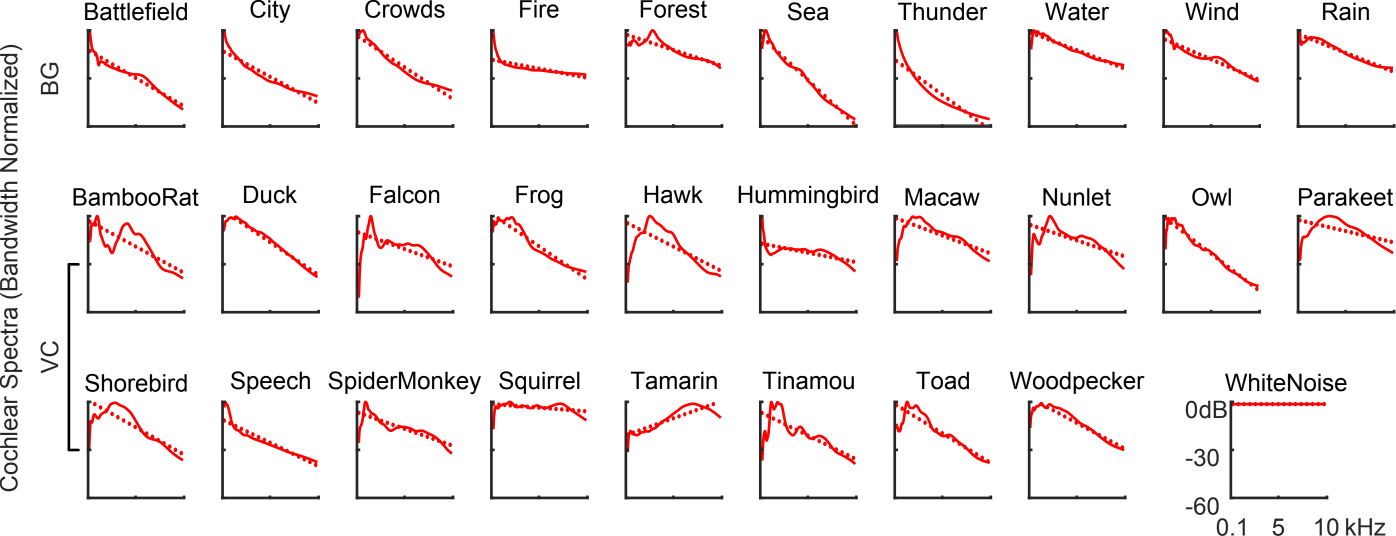

Cochlear Spectra (Bandwidth Normalized)
BG
VC
Battlefield
City
Crowds
Fire
Forest
Sea
Thunder
Water
Wind
Rain
BambooRat
Duck
Falcon
Frog
Hawk
Hummingbird
Macaw
Nunlet
Owl
Parakeet
Shorebird
Speech
SpiderMonkey
Squirrel
Tamarin
Tinamou
Toad
Woodpecker
WhiteNoise
0dB
-30
-60
0.1
5
10 kHz
