# Supplementary material for: Two stages of bandwidth scaling drives efficient neural coding of natural sounds
Source: PLoS Comput Biol. 2023 Feb 14;19(2):e1010862. doi: 10.1371/journal.pcbi.1010862 (PMC9970106; doi:10.1371/journal.pcbi.1010862)
Supplement: Attachment — Submitted filename: ReviewerResponse_Final.docx [file pcbi.1010862.s009.docx]

*Dear Editor and Reviewers*

*We appreciate the detailed comments of both reviewers and have extensively edited the manuscript in order to address all of the comments and criticisms in our detailed response below. We extensively edited the manuscript following the suggestions of the reviewers, provide detailed rationale for our changes, and provide new supporting data analysis to back up our claims. In particular, we have edited the manuscript for clarity and to address the reviewer comments/criticisms. Furthermore, we edited three of the original figures (along with two supplementary) and also added two new supplementary figures. Finally, we also added an author summary section at the request of the editorial board. Once again, thank you for the suggestions, we hope that the resulting changes provide clarification and address all of the concerns.*

Reviewer's Responses to Questions

Comments to the Authors:

Reviewer #1: In “Two stages of bandwidth scaling drives efficient neural coding of natural sounds”, He, Stevenson and Escabi show that the double auditory filter bank performed by cochlea to auditory midbrain performs a whitening operation on natural sounds, yielding neural responses with maximum entropy and thus potentially maximum efficiency. To get to this conclusion, they use a physiologically realistic model of the auditory cochlea and of the modulation filter bank found in the auditory midbrain. The lab is very familiar with the second since they have acquired the neuro physiological data. Overall, the paper is well written, and the results are sound. On the one hand, I was particularly impressed by the clear description of the modulation filter bank and the modulation power spectrum of natural sounds. Just for that part, the paper will be very useful as a tutorial. On the other hand, I have made a list of shortcomings about the analysis and conclusions that need to be addressed – most of them relate to conclusions on optimal representations based on single neuron analyses instead of populations. There are also some important issues in terms of temporal structure that are muddled in the paper and need clarification.

Major Points:

I am not completely convinced by the whitening of the frequency spectrum by the cochlear filters. Yes – it is true that at the level of a hair cell or auditory nerve fiber that gain of the high-frequency channels is higher because the tuning bandwidth is larger. But at the level of the ensemble, there are also more bands (more neurons) in the low frequency range than the higher frequency range. That effect is not well represented in the plots of figure 4. Shouldn’t one consider the density of frequency bands as well?

*RESPONSE: This is an interesting point that we did not initially consider. We agree that the number of neurons / hair cells could certainly impact the amount of total power that is generated by the cochlea across different frequencies. However, after performing additional analysis we are drawing an alternative conclusion to the above.*

*First, in regards to the whitening, note that whitening occurs at the sound ensemble level, not the individual sound level. Figure 4 illustrates the spectrum of individual categories, not the collective spectrum of all the sounds that we used for the study. Indeed, the figure shows that whitening is not necessarily occurring at the individual sound level or even the category level. The figure is intended to illustrate the spectrum variability across the different categories and for the different spectral models. The figure demonstrates that nearly all of the sounds have a decreasing power spectrum for the Fourier model. By comparison, for the cochlear model, the spectrum is far more variable: in some instances it is nearly flat (white), while in other instances it can decrease or increase with frequency (as also indicated by the measured slope distributions, Fig. 5A).*

*The whitening we are proposing occurs only one considers both vocalizations and background sounds. This is more clearly illustrated in Figure 5D (red curve for the cochlear model). The average cochlear spectrum across all sound categories is far more uniform (red continuous curve) than the Fourier model (black) and a simple model based on Gain of the cochlear filters is able to account for this effect (red dotted). We have modified the following section in the discussion for further clarification (~ starting Line 738)*

*“In our case, individual sound ensembles have cochlear spectra that are quite varied and on their own not fully whitened. When considering only vocalizations, the cochlear model outputs overemphasized high frequencies, producing positive spectrum slopes; whereas for background sounds, the cochlear model outputs overemphasized low frequencies, producing negative slopes and, consequently, lower entropies (Fig. 5A,C). Thus, the outputs for vocalization or environmental sounds ensembles individually produced a biased, suboptimal output representation although for both cases they are closer to a whitened spectrum when compared against the Fourier representation. Despite these individual ensemble biases vocalizations and environmental sounds counterbalanced each other and produce combined cochlear spectrum that is, on average, whiter than either category.*”

*As far as the hair cell density, as noted our model currently does not account for this. We looked over the literature for cochlear organization, and contrary to the reviewers suggestion we conclude that there are actually more neurons at high frequencies (when measured on a octave scale), not the alternative as suggested. This clearly sounds counterintuitive, however, it is well known that the frequency organization of the cochlear epithelium follows a nonlinear frequency-position function (Greenwood 1961). Using Greenwoods frequency vs. position function (see below) and the fact that there roughly an even distribution of hair cells per cochlear distance (~100 hair cells / mm over ~35mm of the human cochlear spiral) (Wright et al 1987; see figure panels below), we estimated the distribution of frequencies per octave (which is the spacing we are using for the model) as well as per Hz (which is the spacing for a Fourier transform).*

*(Greenwood 1961)	(Wright et al 1987)*

*If one uses linear spacing, as for a Fourier transform, the hair cell density is skewed to low frequencies following an approximate exponential distribution (as suggested by the reviewer). This is analogous to the scenario described by the reviewer in which there are more hair cells at low frequencies. For octave spacing, however, the distribution is biased towards high frequencies. This occurs, because as can be seen in the frequency position function from Greenwood (plotted in a new figure S4 where we parcellated the cochlear length into ten 3.5 mm segments) there are fewer cells per mm of cochlea at the low frequency end compared to high frequencies. For example, the first octave segment of the cochlea spans ~.8 mm and contains ~80 hair cells while the last octave segment spans ~5 mm and contains ~500 hair cells. Thus, for our modal which has octave spaced filters, the hair cell density would increases with frequency and this in turn would produce an ~8 dB increase in power at the highest frequencies (the opposite of the reviewer’s suggestion) which could provide an additional source of whitening. We now include the following text in the discussion as well as a supplementary figure (S4) to address this issue:*

*“Despite the observed whitening, a reduction of power of ~10 dB is still observed at the highest frequencies for the average cochlear spectrum (Fig. 5D; red). One additional mechanism not explicitly accounted by our cochlear model that could further whiten the cochlear spectrum is the fact that the distribution of hair cells with different best frequencies varies along the cochlear spiral. Our model assumes that frequencies follow octave spacing, yet the cochlear spiral exhibits a nonlinear frequency versus position function spanning 10 octaves for human hearing (20 Hz – 20 kHz) that deviates from an octave approximation at low frequencies [43]. Using Greenwood’s model of the human cochlea [30,43] and the fact that there are roughly 100 hair cells per mm [44] we estimated ~80 hair cells for the lowest octave of hearing (20-40 Hz) and ~500 hair cells over the last octave (10-20 kHz). This change in hair cell density per octave increases systematically with frequency and amounts to more neurons responding at high frequencies. Under the assumption that sound power is integrated by the auditory system across peripheral receptors, this would correspond to an increase in output power of ~ 8dB at the high frequencies (S4 Figure). Thus, such frequency dependent boost in power could potentially further whiten the spectrum of natural sounds.”*

Similarly, the conclusions do not quite jibe with the critical bands obtained in loudness summation experiments. That body of work would suggest that we are performing a low pass filtering on the sounds. I believe you need to address these issues.

***RESPONSE:*** *We revisited the Zwicker, Flottorp, and Steven’s 1957 manuscript on critical bands in loudness summation and based on that data and our interpretation, we do not quite understand the reviewer’s argument. Our interpretation of the study is that for tone complexes or narrowband noise, loudness is roughly constant within a critical band for constant power sounds. Yet outside the critical band the loudness of the tone complex or noise is integrated differently producing an in increases at a rate of ~10 dB /decade. This behavior is demonstrated for various center frequencies, however, the behavior is very consistent regardless of the best frequency they tested (Fig. 2). The curves plotted to illustrate this behavior (Fig. 2 and subsequent plots) do resembles a lowpass filters, however, they are ploting Δf (tone complex spacing or noise bandwidth) versus SPL, not the sound center frequency versus SPL (which is what is needed to demonstrate lowpass behavior). Similarly, although Figs. 11 and 12 resemble lowpass filters, these are plotting the measured bandwidths versus frequency, so technically they are not showing lowpass filter behavior as far as we can tell.*

*From what we gather, the paradigm used for loudness summation in those studies would not be subject to the gain we are proposing and would not produce an increase output for our model. For example, consider the narrowband noise used by Zwicker et al. They varied the noise bandwidth but they maintained a constant SPL while doing so. Our model would produce identical output power for all bandwidths (so long as the noise falls in the filter) because each noise has identical power regardless of their bandwidth. Our model produces a gain because there are more frequencies being accumulated for high frequency filters (because of their larger bandwidth). Thus, for one to observer the behavior we are proposing, one would need to redo these experiments using constant spectrum level noise (as opposed to constant SPL). In such a instance, increasing the noise bandwidth, would increase the amount of energy falling in the filter and one would thus expect an increase in loudness.*

*As a final note, even if the auditory system behaves as a lowpass filter for loudness summation, the behavior may or may not be reflective of the transduction stage and analysis stages we are studying. Specifically, the analysis and model we are using is intended to understand the encoding of detailed spectrotemporal modulations in natural sounds that are much more likely to contribute pitch, rhythm, roughness and timbre. Loudness on the other hand is somewhat of a coarser integration phenomenon, involving long time scales of integration (up to ~250 ms; e.g., Florentine et al 1996) and interactions both within and outside the auditory filters. Thus it likely involves different integration mechanisms than we are describing.*

At a very minimum, you can mention that you are considering optimal principles at a single neuron level and clarify the apparent contradictions that I have raised here.

*RESPONSE: We don’t fully agree on this point, because the entropy measure we are using as an assay of optimality is not a single neuron entropy measure. It is an ensemble entropy across frequency or modulation channels. This is explicit from the equations used to compute entropy (Eqns. 23 and 24) since the equations requires the full distribution of frequency (spectral entropy) or modulation chnanels (for modualtion entropy).*

Related to point 1, one can also imagine estimating ensemble entropy measures that are also take into account the correlations across frequency bands. The results and conclusions might be quite different.

*RESPONSE: We fully agree and are aware that sound correlations can potentially contribute and we actually have several manuscripts that have tried to address the importance of correlated sound structure (Escabi and Schreiner 2002; Chen et al 2013; Sadeghi et al 2019; Xiu et al 2021).*

*While we do acknowledge that the current method is not able to account for dynamically varying spectrogram structure and correlations (i.e., correlations that vary over time as described in Escabi and schreiner 2002; Sadeghi et al 2019), we point out that our measures do account for average correlations across channels, because the modulation power spectrum can account for this information. Note that the MPS is mathematically related to the spectrogram autocorrelation function (Sighn and Theunisen 2003; Escabi & Schreiner 2002) by a Fourier transform so that technically sound correlation information is present in the MPS (i.e., correlation and power spectrum are equivalent although in different spaces). For instance, if a sound has broad spectral correlations the MPS will exhibit dominant energy for low spectral modulations (around 0 cycles/octave). On the other hand, if there spectral peaks at one octave separation, the spectral correlation function will exhibit fluctuations at intervals of 1 octave and the MPS will show a peak at 1 cycle/octave.*

I believe that the same comments (1 and 2) apply to the modulation filtering argument. Again, I agree that midbrain neurons with faster modulations have wider modulation bandwidth tuning (an empirical fact that you have shown !) and that this results in greater gain in that area of the MPS for a single neurons – but there are less of then with this tuning as well – right? (you have that data !).

*RESPONSE: Unfortunately, we don’t have a concrete answer to this and we feel the available data currently do not fully resolve this issue.*

*First, there is evidence from Schreiner and Langner (1988) and our own unpublished work looking at organization (Rodriges et al. SFN abstract 2008) which suggests that spectral and temporal modulation preferences are organized within the IC and that these vary systematically along the medio-lateral aspect of the iso frequency lamina. High modulation frequencies appear to fall at the extremities of the lateral aspect where it is harder to reliable sample the data. The core of the central nucleus is much easier to sample and one could thus potentially bias towards lower or intermediate modulation frequencies if the IC is indeed organized as proposed by Schreiner and Langner. In addition, the neuron density of modulation preferences varies substantially depending on how one analyzes the data which could ultimately bias the interpretation of results. Nearly all of the studies including our own from Rodrigues plotted best modulation frequency distributions using a linear axis. However, it’s very likely that modulation preferences are organized on a log-scale along the tissue within the IC. This would be analogous to the well=known observation that best frequencies in the cochlea are organized approximately logarithmically on the cochlear epithelium. If one were to plot distribution of best frequencies on a linear scale for cochlear fibers, one would conclude that there are far fewer high frequency fibers compared to low frequencies (following an exponential distribution; e.g. below, left panel). Howver, the picture changes dramatically if one uses log-frequency spaced distributions.*

*The idea that best modulation frequencies (BMFs) may follow a logarithmic distribution within the IC is also supported by one of our own studies (Zheng and Escabi 2008) where we plotted measured BMFs on a logarithmic scale (Fig. 4E of that paper). Below, the data is shown both using a linear axis histogram and a logarithmic axis histogram (~0.9 octave spacing). Note that the distribution is far more uniform for the logarithmic axis histogram when compared against the linear axis histogram, which appears far more biased towards low frequencies.*

*Thus we speculate that modulation preferences are likely evenly distributed within the IC, although the distribution is likely for log-modulation freq. (not linear modulation freq.), although additional work is needed to fully resolve this. Theoretically, we fell such a result would be compatible with the bandwidth scaling observation since it would guarantee that the modulation filters provide uniform coverage of the relevant modulation space. Our current implementation follows our observations of the Zheng and Escabi manuscript where modulation frequencies are sampled logarithmically. We’ve added the following to the methods (~Line 190) in order to provide context for the choice:*

*“These primary receptive field parameters determine the modulation tuning of each model neuron and are varied systematically on an octave scale between* $f_{m0}=$*-512 to 512 Hz (0.25 octave steps) and* $\Omega_{0}$*=0.1 to 3.6 cycles/oct (0.1 octave steps). We choose octave spacing for these primary receptive field parameters because both mapping and modulation processing studies [27,28,36] indicate that modulation preferences are roughly evenly distributed when plotted on an octave scale.”*

I appreciate the focus on the cochlear representation, followed by the analysis of the midbrain representation. But the picture of auditory periphery processing is incomplete. Low-frequency auditory nerve fibers also phase lock to the actual waveform of the signal in the narrowband filtered signal, providing additional information that could further increase spectral resolution and the detection of voice pitch. The temporal structure you describe (e.g. in the discussion in lines 800-807) results for the high frequency cochlear filters and the corresponding fast amplitude modulations captured by your model. The correlated phase locked activity at low frequencies clearly also carries information on fast time structure. By the way this is the sTFS that is discussed in the paper that you cite in [61] in the context of your work that discusses fast temporal modulations. I think this could lead to further confusion on this temporal coding topic that is already poorly understood by many. It is possible that the sensitivity to phase for low frequency AN fibers end up contributing to the high temporal modulations sensitivity that you are describing but that is not part of your auditory model.

*RESPONSE: Thanks for this point, we have revised the discussion to clarify this and avoid confusion. As noted, our model is specifically designed to extract and to explore the representation of amplitude modulations. We choose this intentionally, in order to focus our attention on how amplitude modulation information represented in the central auditory system. The model does not explicitly extract the fine structure, as noted, because it employs a Hilbert transform which is designed to extract the sound envelope. As part of a new project looking at speech and vocalizations in noise, we are currently using and exploring a rectifying stage (as opposed to a Hilbert transform) within the cochlear portion of the model, which incorporates fine structure. We agree that the distinction between temporal envelopes and fine structure is could be confused and for this reason we have removed “fine structure” and replaced it with “temporal information” to avoid confusion.*

This is somewhere between a major and minor point. As you know, prior computational papers that have addressed optimal coding strategies (and verified Barlow’s hypothesis) have often started from an objective function (e.g. it would be entropy here) that they then maximize to find the best set of filters (as done by Lewicki for example using a sparsity objective function). Your approach is a bit different in that you just examine a biologically inspired model and show that it performs whitening. This is clearly interesting but maybe not quite as powerful. It might be too much to ask to try to find the optimal double filter bank but you should probably discuss this more clearly and think about how one might do this.

*RESPONSE: We agree that the approaches differ, however, we feel that each has their strengths and weaknesses. For example, even though such prior studies have derived optimal filters based on a theoretical principles, even these require assumptions about the nature of the code and the optimization strategy itself. Lewicki 2002, for instance, optimized a linear basis set to maximize the likelihood of statistical priors of natural sounds and in 2006 (Smith & Lewicki) they then tested a sparse objective. On the one hand, the linear basis set used ignores basic nonlinearities important for envelope extraction, which are known to exist physiologically and our model partly achieves with a Hilbert transform and subsequent lowpass filtering. Also, why not optimize the basis set for a behavioral task objective? That seems far more relevant biologically that an objective based on reconstruction quality. And finally, even though such approaches may give insights into optimal filtering strategies they don’t full explain “why” the filters are optimal. By comparison, we started with the known biological constraint and tried to mechanistically tease apart it’s possible role for encoding (the why), which we feel is important and equally valuable.*

*The idea of finding a double filterbank is actually quite interesting, this is something that we have considered although not implemented. Including such a result would be a monstrous task and we feel this is a subject a different manuscript. We’ve added the following to the discussion to address these points:*

*“Our approach differs from various prior studies which have derived optimal basis sets for representing natural sensory stimuli and testing the efficient coding hypothesis [23,24,49,50]. Although these studies employ a rigorous framework to test a computational theory, they nonetheless require assumptions about the nature of the proposed code and the optimization strategy used. For example, these models often assume linear basis sets which don’t account for nonlinear characteristics of neural processing and often employ objective functions that are not biologically driven. More recent studies have overcome such limitations by employing deep neural network and behaviorally guided objective functions for optimization, which are presumably more biologically relevant [51,52]. Nonetheless, such models often have tens of thousands of parameters and can be difficult to interpret mechanistically. In our case, rather than optimizing a model, we employed a model with known biological constraints to develop a mechanistic explanation of the acoustic representation. This allowed us to demonstrate how sound whitening is achieved for multiple auditory features across multiple levels of auditory processing. In future studies, it would be valuable to derived jointly optimal multi-stage filterbank in order to further identify optimal strategies and mechanisms for natural sound processing.”*

Minor Points:

The introduction does a very good job at summarizing what is known about natural sound statistics and auditory representations and also at introducing the modulation power spectrum. It falls a bit short when introducing the question addressed in the paper in the last paragraph.

*RESPONSE: We agree. We have edited this last paragraph in order to more clearly introduce the study objective and the question we propose to study.*

457 sound segments is probably more than sufficient but one will notice that it is much less than the typical number of images used by researchers investigating visual object representation.

*RESPONSE: There are clearly recent studies that have used machine learning and deep neural networks to study auditory and visual representations and these have used much larger databases than we are using (e.g., Kell et al 2018; Yamins et al 2014). This is in large part due to the fact that deep neural networks have a huge number of parameters and training them consequently requires extensive amounts of data in order for them to generalize. However, we are not training such high-dimensional models and the number of sounds we used and their durations is actually in line and exceeds those of prior studies that have studied sensory signal statistics. For instance, below is a summary of several databases used for several prominent auditory and visual images statistics studies:*

| Study | Types of database | Total duration |
| --- | --- | --- |
| Attias and Schreiner 1996 | Vocalizations and environmental sounds | 2 min |
| Lewicki 2002 | Speech, animal vocalizations and environmental sounds | Environmental = 45 s  Speech = 100 utterances of unknown duration  Vocalizations = 45 vocalization of unkown duration |
| Nelken et al. | Vocalizations and environmental sounds | 21.6 min |
| Rodrigues et al. 2010 | Speech, animal vocalizations and environmental sounds | 176 min |
| Sighn & Theunissen | Zebra Finch Song, Speech, environmental | Zebra finch = 40 s  Speech = 40 s  Environmental = 45 s |
| Sadeghi et al 2019 | 13 categories and 15 sounds / category | 195 sounds totaling 32.5 min (10 sec per sound) |
| Ruderman and Bialek | Natural visual scenes | 45 images |
| Olshausen | Natural visual scenes | 10 images |

*For our study we are using 457 sounds from 29 distinct categories with a total duration of 4.8 hours. This far exceeds the database size both in duration and number of samples of all these prior auditory and visual studies. The parameters used for the auditory model have already been established based on prior physiological studies and require no training. Thus, based on our own experience as well as the sample sizes used prior studies computing similar statistics (power and modulation spectrum) we are confident that this is a reasonable sample size.*

L 463. Since you are talking about cochlear filters it is a bit weird to add at the end of this sentence “..analogous to cochlear filter tuning”. I know you mean model filters are analogous to the actual physiological filtering, but I bet that many readers will get stuck here.

*RESPONSE: removed.*

In figure 2C and in the text l457-476, you might also want to discuss the gain of the filters for different frequencies. The amplitude of the impulse response in the top panel of 2C looks identical for different frequencies but then in the color matrix representation one can clearly see a high pass filtering. This needs an explanation here.

*RESPONSE: Thanks, we have added the following clarification to the figure legend.*

*“In the time domain, the cochlear filter impulse responses (C) have frequency dependent peak amplitudes and delays and the impulse response durations scale inversely with frequency. For visualization purposes and to allow for ease of comparison the impulse response line plots for the three examples are normalized to a constant peak amplitude (C, top).”*

*We have also edited the description in the results section to clarify this.*

Results. L 513-520. To better describe the structure of the speech cochleogram, I would annotate the figure to clearly show that the first bands of energy harmonics that are due to the voice pitch, that the ones found in the middle are the formants and that there are then vertical bands in the higher frequencies that correspond once again to voicing (as you mention in the text). Also your text description should mention formants. Personally, I find them more visible in the spectrograms with df=10 or 30 while they appear compressed in a small range in the cochleogram.

*RESPONSE: Agreed. We have edited figure 3 and related text and legend. We’ve added colored panels to a segment of the speech that covers different frequency bands in order to highlight these features and more accurately describe them.*

Related to 5. In the discussion on the role of temporal vs spectral modulation (~l788-800) you focus on the detection of pitch. Here again one might want to talk about speech formants as well.

*RESPONSE: Thanks for this suggestion, we’ve modified the paragraph as follows:*

*“Spectro-temporal modulations are also critical for speech perception, contributing to various perceptual attributes such as voice quality and pitch, vowel and consonant identification, phonetic and word segmentation and, ultimately, speech recognition or discrimination abilities. As demonstrated, different spectro-temporal decompositions accentuate a unique set of spectro-temporal features which produce distinctly different spectro-temporal outcomes. Thus, the unique differences in Fourier based versus cochlear representations can ultimately lead to different interpretations of the cues that are important* physiologically and perceptually*. For instance, formants show up as relatively coarse fluctuations in power across frequency that are are visible in both the Fourier based and cochlear representation. In both instances, these show up in modulation filters with low spectral modulation, yet they appear more compressed in the cochlear model do to octave spacing. Voicing pitch on the other hand, is even more dramatically impacted by the spectro-temporal representation. When speech is analyzed using narrowband equal resolution Fourier based filters, temporal modulations are severely limited (<50 Hz) while voicing harmonic content (spectral modulation) related to pitch is accentuated [1]. This harmonic content is a critical cue for voice quality and gender identification. In contrast to conventional spectrograms, the cochlear model only extracts a few harmonics for the low frequency range of hearing, yet it accentuate temporal information at the high frequencies. Such frequency dependent transformation is likely critical for perception and coding of speech and perceptual models need to consider such differences in the sound representation…..”*

Reviewer #2: The authors compare multiple representations of a set of natural sounds. One of the representations is matched to encoding properties of the auditory processing pathway, as inferred from neural and perceptual experiments. In particular, it makes use of a two stage filter in which each stage uses bandwidth scaled filters (the filter bandwidth increases with the center-frequency). The other representation is standard in audio signal processing techniques and uses constant bandwidth filters in both stages. The authors demonstrate that the biologically inspired "bandwidth-scaled" representation is more efficient (i.e., higher entropy representations of natural sounds) than the standard audio representation.

Although, it is not a new idea that perceptual systems are optimized to efficiently represent natural stimuli, this work is a novel and worthy contribution to that field of work. The logic of the paper seems sound, the methods are appropriate, and the results support the conclusions. Given this, I believe this paper is of interest to the scientific community and merits publication.

I find no major flaws and I will devote the rest of this review to suggestions and comments for the authors to consider. Three of them I think are quite important. The rest are minor.

*RESPONSE: Thank you, we appreciate the positive assessment of our work.*

+ Important comment 01: It was only on reaching the paper discussion that I realized I wasn't sure what the authors meant by "bandwidth scaling". Given its prominent usage in the title and abstract I think it should be defined more clearly early in the paper and continually emphasized throughout.

In the abstract it is stated that: "bandwidth scaling produces a frequency-dependent gain that counteracts the tendency of natural sound power to decrease with frequency, resulting in a whitened output representation." I find this phrasing confusing. The "gain" results from the "larger bandwidth" pooling signal over a larger range of frequencies. This is more than just a gain as it extracts different features from the audio waveform.

In addition, the title of the paper references "two stages of bandwidth scaling" but the definition in the abstract only references the first stage.

I assume the title refers to the fact that in both stages the filter bandwidths grow larger as the center-frequency increases. This should be stated explicitly. If the term means something more specific than what I have just mentioned, that should be stated. Either way the definition should be prominent in the paper and should clearly reference both stages of the processing heirarchy. Otherwise the title should be modified.

RESPONSE: *The reviewer’s assessment is correct and we have edited the abstract to more clearly define bandwidth scaling early on in the manuscript. We’ve also made changes to the introduction (lines 91-93) in order to further clarify this point.*

+ Important comment 02: In both the abstract (lines 30-32: ) and in the discussion (lines 792-793) the authors state that cochleagrams "sacrifice spectral information while producing a more robust temporal representation" or "accentuate temporal information" relative to short-time Fourier Transform (STFT). I find this phrasing confusing. A STFT can easily accentuate a temporal information by using shorter windows. There is nothing inherent in an STFT that favors spectral resolution over temporal resolution. I see the major difference between the two as: STFT uses the /same/ spectrotemporal resolution for all frequencies (arbitrarily so); whereas the cochleagram representation favors better spectral resolution in low-frequencies and better temporal resolution at high-frequencies (for good reasons, as these results show). Although, this is a more subtle distinction, I believe it is more accurate, and I suggest the authors state something like this in both the abstract and discussion. A clear and succinct definition of bandwidth-scaling will aid this, as it is precisely the bandwidth scaling that cochleagrams (and midbrain modulation spectral representations) have that Fourier Transforms and conventional modulation power spectra lack.

*RESPONSE: Agreed, the reviewer is correct and we have edit this statement in the abstract for correctness:*

*The abstract now reads: “Whereas Fourier-based spectrographic decompositions have constant time-frequency resolution at all frequencies, cochlear filters exhibit bandwidth scaling, such that filter bandwidths increases with characteristic frequency. This systematic increase in bandwidth produces a systematic decrease in spectral resolution and increase in temporal resolution with increasing frequency.”*

*We have also added the following to the discussion: “Both peripheral and mid-level auditory structures have scale-dependent and spectro-temporally compact filters, analogous to a multi-dimensional wavelet decomposition of sounds. These filters differ from conventional Fourier representations, which lack scaling and have constant spectro-temporal resolution.”*

+ Important comment 03: the authors state in the submission that code for their auditory model is available via GitHub. I searched both the main text and supplemental for a URL but did not find one. A link to the code repository will be very helpful.

*RESPONSE: Thanks, we’ve added the information for the GitHub repository.*

+ minor comment: the authors have not demonstrated that the biologically inspired representation is an "optimally efficient" representation, only that it is more efficient than more broadly known representations (Short-Time Fourier Transform and Modulation Power Spectrum). I would be curious to know how the representation entropy changed with more subtle changes in representation (e.g. what if the empirically determined constants in Equations (3), (7) etc. were altered to create a /different form of bandwidth scaling/? What if the number of cochlear bins were altered? How does the sparse coding representation Lewicki [Ref 23] compare?). This is not a small request, and a full answer perhaps deserves a separate paper. But perhaps this question could be raised in the discussion?

*RESPONSE: We have edited the following paragraph in order to clarify how our results compare to those of Lewicki:*

*“At the cochlear level, our results indicate that bandwidth scaling is matched to the power spectrum statistics of environmental sounds and vocalizations. This is consistent with previous work from Lewicki [23,25] showing that the optimal filters for representing natural sounds are depend on the stimulus categories used during training and that compact filters resembling those in the cochlea are obtained only when both vocalizations and environmental sounds are included. In our case, individual sound ensembles have cochlear spectra that are quite varied and on their own are not fully whitened. When considering only vocalizations, the cochlear model outputs overemphasized high frequencies, producing positive spectrum slopes and indicating that the bandwidth scaling overcompensates the power spectrum trend. By comparison, for background sounds, the cochlear model outputs overemphasized low frequencies, producing negative slopes and, consequently, lower entropies (Fig. 5A,C). Thus, the outputs for vocalization or environmental sounds ensembles individually produced a biased, suboptimal output representation although for both cases they are closer to a whitened spectrum when compared against the Fourier representation. Despite these individual ensemble biases vocalizations and environmental sounds counterbalanced each other and produce combined cochlear spectrum that is, on average, whiter than either category.”*

*As for changing the types of scaling, this would be quite tedious to do especially since we would need to do this both in the cochlear and midbrain stages to fully understand the repercussions of the different scalings (which would require changing the relationship across two dimensions), so indeed this would likely be something to consider for a separate manuscript. However, based on the current results which show that the entropy is near 1 (the theoretical maximum), we predict that changing the frequency vs. bandwidth scaling relationship of the filters (by either making the rate of change less or more gradual) would necessarily decrease the overall entropy.*

*Finally, regarding the number of cochlear bins used, this does not affect the computed entropy substantially so long as the spectrum is not undersampled. This is the case because the entropy equation we are using (Eqn. 23) is normalized for the number of bins used and is always between 0 and 1 (normalized by log2(N), which is the theoretical maximum entropy that can be attained given a chosen number of bins). To illustrate this, we computed the spectral entropy for speech while varying the number of bins (below). Note the spectral entropy asymptotes to an approximate constant value. In our case, we are using 664 bins which should suffice and is approaching the asymptote.*

+ minor comment: another issue that would be of interest to many readers is how this compares to studies of other perceptual systems. Is it known that the visual or olfactory system use such "whitened" representations. Is an analogue of bandwidth scaling involved? If so, pointing readers to the relevant papers would be helpful. If not, the authors might remark upon this.

*RESPONSE: As far as we are aware, whitening in other modalities is likely mechanistically different. For instance, in vision, whitening of spike trains has been shown for individual neurons whereas we are dealing with whitening at the ensemble level. We’ve added the following to the discussion:*

*“In addition, the observed whitening is likely mechanistically different from whitening in other sensory modalities and may be unique to audition. For instance, although multiple levels of whitening are observed in the visual system the known mechanisms differ from those described here. For instance, whitening of visual scenes in the lateral geniculate nucleus is achieved by temporal decorrelation of the spike trains that occurs at the individual neuron level and which is restricted to low frequencies (<15 Hz) [53]. In primary visual cortex, additional whitening is achieved through nonlinear interactions of the classical and nonclassical receptive fields of individual neurons, which again are restricted to low frequency information (<36 Hz) [54]. In our case, whitening is an ensemble level phenomena that involves multiple tuned filters and which involves temporal information exceeding several hundredths of Hz.”*

+ minor suggestion: Table S1 would be more interesting if it included columns for the entropies of each representation. I would be curious to see which sounds are outliers in this representation scheme. Another way to show this could be to simply print the cochleogram/spectrogram entropy value by each subplot in Fig 4, and the midbrain/MPS entropy value by each subplot in Fig 7.

*RESPONSE: Agreed, we’ve added the entropy measurements as suggested above the figure panels in both Fig. 4 and 7.*

+ minor suggestion: Fig 7s A-C all use the same colorscale, which means figs 7C look boring to the eye. This clearly makes the point that the midbrain inspired representation is "whitened" relative to the modulation power spectrum. But it would be interesting to see these plots on an adjusted colorscale that shows their structure more clearly. Perhaps a supplementary figure? Or an additional subplot 7D?

*RESPONSE: Agreed. We have added a new supplementary figure (S3) that now shows the Midbrain Model results in more detail using a compressed and individualized colorscales for each sound.*

+ minor suggestion: Line 208 ("Similarly, perceptually measured modulation bandwidths in human listeners scale with modulation frequency [25,26].") I find confusing. The modulation bandwidths of the auditory system are /inferred/ from perceptual thresholds (it is these that are /measured/).

*RESPONSE: Agreed, we have reworded the sentence for clarification as follows:*

*Similarly, human modulation bandwidths, which are derived using perceptual measurements, also scale with modulation frequency [25,26].*

+ minor suggestion: at Line 244, I expected to read about how Modulation Power Spectra were computed for the Fourier spectrographic decompositions. This would mirror the earlier structure describing how the biologically inspired heirarchical representations were constructed. I would suggest moving the section on "Spectro-temporal resolution and uncertainty principle" to after the section on "Modulation power spectrum (MPS)".

*RESPONSE: Agree. We’ve moved this section as suggested.*

+ minor suggestion: Line 280. I was initially confused by what was meant by the "two biologically inspired sound decompositions" and had to read several times before I realized the two levels of the heirarchical decomposition were being discussed as two separate decompositions. I would suggest the authors be mindful of this and re-word the paragraph.

*RESPONSE: We have reworded this sentence as follows:*

*“Here we describe the calculation of the MPS at multiple levels of the auditory system model using the 1) cochlear model and a 2) midbrain model decomposition as well as for the reference 3) Fourier based spectrographic decomposition.”*

+ minor suggestion: Lines 286-289: I don't think the technical details of how Singh and Theunissen computed the Fourier MPS relevant. This is complexity that will tax a reader but doesn't help them understand what is being done here. I would suggest removing these sentences and cutting straight to Eq. (15). A simple citation of Singh and Theunissen here will give them due credit.

*RESPONSE: We have removed this description and streamlined the writing while keeping the refence as suggested.*

+ minor suggestion: lines 432-433: "In practice, the selection of these ranges has a minimal impact on the entropy calculation and does not account for the entropy differences between sounds.". This is too technical for the main text but some readers might appreciate a supplementary figure showing this.

*REPONSE: We reworded this sentence to clarify our intended meaning. Essentially, we are trying to point out that the range for the auditory midbrain calculation produces very similar entropy values as the other representation for white noise. The statement now reads:*

“This upper limit for the auditory midbrain representation did not bias the entropy calculation, since all representations have a comparable entropy for white noise (Fourier: 0.95, 0.95, 0.95 for $\Delta f$=30, 120 and 480 Hz, respectively; Cochlear: 0.95 bits; and Midbrain: 0.93).”
