# Supplementary material for: Two stages of bandwidth scaling drives efficient neural coding of natural sounds
Source: PLoS Comput Biol. 2023 Feb 14;19(2):e1010862. doi: 10.1371/journal.pcbi.1010862 (PMC9970106; doi:10.1371/journal.pcbi.1010862)
Supplement: Attachment — Submitted filename: ReviewerResponse_R2_Final.docx [file pcbi.1010862.s010.docx]

*Reviewer #1: Dear authors,*

Dear Frederick, Thanks for meeting with us and taking the time to discuss the below comments in detail. As noted below, we’ve added the suggested analysis and also have edited the manuscript at several key locations in order to address the concerns and provide the needed clarification.

*Thank you for your detailed answers to my first round of reviews. I am mostly satisfied and I am glad that your manuscript now also mentions the density of hair cells in the cochlea and the fact that it is not perfectly logarithmic at low frequencies but a bit more linear (at least in humans). However, I believe that you did not completely understand my point relating to the power gain – and I was probably not completely clear. I am not disagreeing that there is a whitening in the frequency power curves (and as you describe well this is on average, etc). Clearly this is true because higher frequency auditory fibers have larger tuning bandwidth and thus, they integrate over a larger frequency range. Since (on average) natural sounds have less energy in the high-frequency range, the result is a more equal distribution of power response per unit. And more uniform distribution yields a higher entropy. This is the main message of the first part of the paper and it is well done and convincing. In addition, you perform this calculation for two levels of processing by including the modulation tuning; I very much appreciate that effort. It yields a nice and complete picture.*

*However, I think that one has to be more careful when talking about power, gain and the slopes (your analyses in figures 4 and 5). More precisely, a frequency power spectrum has units of density: power per frequency. One can plot that power density using linear or log units in the x axis. When one does a cochleogram and then estimates power, you get an “equal sampling” in log units – so now your power density curve is in power per octave. You could still plot that in linear or log frequency units but the curve means something else that the power per frequency. For example, when you compare power in Fourier Spectral and Cochlear Spectral, you can either compare two different densities in power/f vs power/logf units as you have done in figure 4 or attempt to compare densities in the same units. If you did this by transforming the power/logf obtained with the cochlear filter into a power/f, you would get a boost in the low frequencies. Think of your 100 cochlear filters distributed in log scale (as in 100 red dots in Fig.4) – now you resample these into 100 bins in linear scale by summing or splitting the energy as needed (but not by taking the average). You will then obtain a density in power/f. And you will also see that the low f are boosted once again.*

I think that this difference explains the discrepancy between the “whitening” that maximizes the “lifetime” spectral entropy across units and the experiments in loudness, frequency discrimination, etc that suggest the oversampling in the lower frequency range. And I also think that for the power/gain argument, it only makes sense to compare curves that have the same units. You could then clearly distinguish the entropy argument (a uniform sampling) with the power argument. If you were able to clearly explain this in your paper, I think that it would have a greater impact.

**RESPONSE:** We certainly agree that power in the vast majority of sounds dominates the low frequency range of hearing and likewise perception of many sounds and phenomena are likewise dominated by low frequency hearing. The reviewer is correct that mechanism we are proposing does not explicitly boost the power of natural sounds at high frequencies through a gain mechanism. Instead, we are proposing that high frequencies neurons sacrifice spectral resolution in order to accrued more power. This whitens the output of the cochlea, not the natural sound itself, and ultimately produces a vastly different representation that would be obtain with more conventional filterbanks (as demonstrated in the MS).

We have performed several changes to clarify this point explicitly in the manuscript and to address the above concern:

a. We added two new supplementary figure which compares the power distribution of the cochlear model using matched linear spacing and units (power per Hz) as suggested. The figure illustrates that the power in the cochlear model output per units of Hz (Fig. S5) does indeed follow a very similar trend the conventional Fourier spectra (as suggested). In addition, the slope distributions for this normalized form of the cochlear spectrum (Fig. S6 ; in dB/Hz) does indeed behave nearly identically slope distributions as observed for the Fourier spectrum (Fig. 5A). Together, this shows that the power distribution on the cochlea per Hz is indeed similar to that of the incoming sound (as suggested). The primary distinction at the output of the cochlear model is that the integrated power across filters with variable bandwidths produces an output that is roughly whitened, which is the main result of Fig. 5 and 6. Together with Fig. 6, the supplementary analysis demonstrates that the bandwidth scaling is the principle mechanism responsible for the cochlear output whitening.

To further add context, we have edited the text at several key locations to make these points and the distinction between whitening of the sound power and whitening of the neueral response explicit and clear:

b. Where possible we have strategically changed our wording to “output whitening” or “cochlear spectrum whitening” in order to differentiate it from whitening of the sound itself.

We have also added several sentences to provide clarification for the points noted above:

c. Line 109-11 in the introduction
d. Line 576-578 in the results
e. Line 619 -620 in the discussion
f. Lines 752-758 in the discussion

*I am happy to discuss this point with you so that we can reach an agreement. (Alternatively, I could write a comment to your paper).*

Thanks for meeting with us. We initially did not have a clear picture of the criticism and the meeting helped clarify it.

*Minor points.*

*1. As I was rereading your paper, I wonder to what extent equations 22 and 23 depend on N. Clearly if the numerator of 23 goes as logN then the exact number N does not matter. I am suspecting that as N gets large enough this is the case. Did you check whether you were in that regime?*

**RESPONSE:** Yes, we did check that this is the case. Essentially, it is required that we have enough samples along the spectral axis (N) to assure that that spectrum distribution is adequately sampled. We actually demonstrated this in our prior response to reviewer #2, who had a similar concern about a possible dependence on N. Below is our response, with an example showing how the speech cochlear entropy varies as function of N. As you can see, the estimated entropy for the number of channels we are using (N=664) approaches a near constant value, consistent with the idea that we are approaching the asymptotic limit.

“Finally, regarding the number of cochlear bins used, this does not affect the computed entropy substantially so long as the spectrum is not undersampled. This is the case because the entropy equation we are using (Eqn. 23) is normalized for the number of bins used and is always between 0 and 1 (normalized by log2(N), which is the theoretical maximum entropy that can be attained given a chosen number of bins). To illustrate this, we computed the spectral entropy for speech while varying the number of bins (below). Note the spectral entropy asymptotes to an approximate constant value. In our case, we are using 664 bins which should suffice and is approaching the asymptote.”

*2. In Fig 5B, I would have axis labels at 0.5 and 1.0 instead of 0.6 and 1.1. 1.0 is the maximum value of these scaled entropy and it makes more sense to be very explicit about that.*

**REPSONSE:** Fixed.

**Additional Edit:** Finally, in addition to the above changes, we noticed that upon moving the description of the MTF to the supplementary text during the last revision, there was no longer a definition of the MTF in the current version. We thus edited the text surrounding equation 19 (Lines 351-356) to provide the necessary information.

*Reviewer #2: I am satisfied the authors have addressed all issues I raised.*

Thank you!
